# The Impact of D2 Versus D1 Lymphadenectomy in Siewert II Gastroesophageal Junction (GEJ) Cancer

**DOI:** 10.1245/s10434-024-15623-z

**Published:** 2024-07-30

**Authors:** Nathan J. Alcasid, Deanna Fink, Kian C. Banks, Cynthia J. Susai, Katherine Barnes, Rachel Wile, Angela Sun, Ashish Patel, Simon Ashiku, Jeffrey B. Velotta

**Affiliations:** 1 Department of General Surgery, University of California, San Francisco-East Bay, Oakland, CA USA; 2 Division of Research, Kaiser Permanente Northern California, Oakland, CA USA; 3 Division of Thoracic Surgery, Department of Surgery, Kaiser Permanente Northern California, Oakland, CA USA

**Keywords:** Lymphadenectomy, Gastro-esophageal cancer, Minimally invasive esophagectomy, Gastro-esophageal junction, Staging

## Abstract

**Background:**

Although multiple treatment options exist for gastroesophageal junction (GEJ) cancer, surgery remains the mainstay for potential cure. Extended nodal dissection with a D2 lymphadenectomy (LAD) remains controversial for Siewert II GEJ cancer. Although D2 LAD may lead to a greater lymph node harvest, its effect on survival remains elusive. The authors hypothesized that additional D2 dissection in Siewert II GEJ cancer does not lead to increased survival.

**Methods:**

This study reviewed Siewert II patients who received a D1 or D2 LAD in addition to minimally invasive esophagectomy (MIE) after receiving neoadjuvant chemoradiation or perioperative chemotherapy (2012–2022). The patients were followed for up to 5 years. The outcomes measured were survival, number of nodes sampled, and operative time. The association between D1 or D2 LAD and overall survival was analyzed with Kaplan-Meier methods and a multivariable Cox regression model.

**Results:**

Among 155 patients, 74 % underwent D1 and 26 % underwent D2 LAD. The patients with D2 had more than 15 lymph nodes harvested more frequently than those who had D1 (83 % vs 48 %; *p* < 0.001), with no difference in positive nodes (2.8 ± 5.2 vs 2.1 ± 4.2; *p* = 0.4). The patients with D2 LAD had a longer median operative time than those who with D1 LAD (362 vs 244 min; *p* < 0.001). In Kaplan-Meier and multivariable Cox regression models, overall survival did not differ significantly between the patients undergoing D2 and those who had D1 (adjusted hazard ratio [aHR], 0.52; 95 % confidence interval [CI], 0.25–1.00; *p* = 0.067).

**Conclusions:**

Little consensus exists regarding the optimal lymph node harvest for GEJ cancers. In Siewert II cancer, D2 LAD may not be mandatory and may lead to increased operative morbidity with no significant difference in survival.

**Supplementary Information:**

The online version contains supplementary material available at 10.1245/s10434-024-15623-z.

Despite ongoing advances in multimodal treatment options for gastric and esophageal malignancies, mortality rates remain high.^1^ During recent decades in the United States, cancer of the gastroesophageal junction (GEJ) has presented as the most common form of both gastric and esophageal malignancies, with an almost sevenfold increase in incidence.^1,2^ Although multimodal treatment has evolved to include a combination of chemotherapy and/or radiotherapy, surgical resection remains the mainstay for potential cure.

The Siewert classification system was created to define GEJ tumors anatomically based on their distance from the GEJ, with both diagnostic and therapeutic implications.^1^ Siewert I-type tumors are located with a tumor epicenter ranging approximately within 5 cm proximal to the GEJ. Siewert II tumors have an epicenter at the GEJ, whereas Siewert III tumors are located with a tumor epicenter ranging within 5 cm distal to the GEJ.^3^

Tumor location also has implications regarding nodal metastasis, with Siewert I tumors typically involving upper mediastinal lymph nodes and Siewert II/III tumors more frequently involving lower mediastinum in addition to the intraabdominal celiac axis.^4^ During the past decade, more than half of GEJ tumors have been diagnosed as Siewert II.^5^

With these anatomic definitions in place, the tumor-node-metastasis (TNM) staging classification of the eighth edition of American Joint Committee on Cancer (AJCC) defines Siewert II tumors as esophageal tumors, but no consensus guidelines regarding the optimal surgical approach exists, whether an esophagectomy or total gastrectomy.^6,7^ Additionally, the extent of lymphadenectomy (LAD) during definitive surgical resection also is controversial. The Japanese Gastric Cancer Association defines the extent of LAD as D1 (containing the peri-gastric lymph node [LN] basins) or D2 (with LN harvesting beyond the peri-gastric LNs and along the celiac axis).^8^ Worldwide differences exist, with more D2 LADs commonly identified in the Eastern hemisphere.

The unpopularity of D2 LAD lies in its more technically demanding approach associated with higher perioperative morbidity and no evidence of improved overall survival compared with D1 LAD.^8,9^ Although D2 LAD recently has been found to have lower locoregional recurrence and gastric cancer-related deaths than D1 LAD, these benefits have been seen for gastric malignancies but are not well-described for GEJ tumors.^10^

The current National Comprehensive Cancer Network (NCCN) guidelines recommend 15 lymph nodes for an accurate gastric cancer harvest, and although findings have shown that a larger nodal harvest is linked to favorable overall survival in purely esophageal and gastric carcinomas, these data have not been well-evaluated for patients with Siewert II tumors.^8,11^ Although D2 LAD may lead to a larger lymph node harvest, its effect on overall survival in GEJ remains elusive. We hypothesized that additional D2 dissection in Siewert II GEJ cancer does not lead to increased survival and may not need to be routinely performed.

## Methods

### Study Population

We performed a retrospective review of all adult patients (age, ≥ 18 years) with Siewert II GEJ cancer from 1 January 2012 through 30 June 2022 within our integrated health care delivery system, which serves as a tertiary referral center. Patients with Siewert I and III GEJ tumors and those with Siewert II GEJ tumors who did not undergo surgery were excluded from the study. Baseline characteristics, comorbidities, and cancer stages of patient’s with Siewert II GEJ cancer who underwent a D1 LAD were compared with those of patients who underwent a D2 LAD.

This study was approved as a retrospective, observational, and data-only cohort study by The Kaiser Foundation Research Institute’s institutional review board, with a waiver of consent because of minimal risk with de-identified data

### Study Design

At our institution, the diagnosis of patients is performed with a combination of computed tomography (CT), positron emission tomography (PET), and endoscopic ultrasound fine-needle aspiration (EUS-FNA). The diagnosis then undergoes multidisciplinary tumor board review. Our Siewert GEJ classification defined Siewert I type tumors as those located with a tumor epicenter ranging approximately within 5 cm proximal to the GEJ, Siewert II tumors as those with an epicenter at the GEJ and up to 2 cm past the GEJ, and Siewert III tumors as those with a tumor epicenter ranging within 5 cm distal to the GEJ.^3^ These tumors were defined using the aforementioned criteria by an advanced gastrointestinal endoscopist upon EUS evaluation. We did not define or classify tumors based on the esophagogastroduodenoscopy (EGD) at the time of the initial biopsy.

Surgical resection was performed primarily via a minimally invasive Ivor-Lewis approach for 146 of 155 patients, with a minority of the patients undergoing the trans-hiatal esophagectomy approach. The cases were managed by a select group of 10 thoracic surgeons in three high-volume centers of excellence with minimal differences in regional practices. The main determination for performing a D1 rather than a D2 LAD was based on a combination of individual surgeon preference and multidisciplinary tumor board discussions about various perioperative approaches for either systemic chemotherapy or a combination of neodjuvant chemotherapy and/or radiotherapy.

Management of the tumor varied largely based on the discretion of the oncologists present on the tumor board and on their decisions at presentation of the tumor characteristics at the time of preoperative evaluation, such as number and degree of intraabdominal lymphadenopathies seen on PET scan and the presence of gastrohepatic lymphadenopathy on EUS. Extension of a D2 LAD was defined as nodal tissue harvested beyond the peri-gastric tissues and extending into the celiac axis. Splenectomies were not performed.

At our institution, the standard protocol for D2 LAD entails the addition of a separate gastrointestinal surgical oncologist or foregut surgical team from the thoracic surgery team. We chart-reviewed all patients with a diagnosis of Siewert II tumor to validate eligibility and clinical stage and to collect data on the LAD (including D1 vs D2, anastomotic leak status, blood loss, number of nodes sampled, and number of nodes positive), as well as the clinical history including prior diagnostic laparoscopy, neoadjuvant and adjuvant radiation and chemotherapy, and pathologic stage. Age, race/ethnicity, sex, body mass index (BMI) at diagnosis, Charlson Comorbidity Index (CCI) score, smoking history, history of alcohol abuse, histology, and operation time during the LAD were obtained from electronic databases.

### Outcomes

The primary outcomes measured were overall survival from the date of diagnosis until up to 5 years, number of nodes sampled, number of positive nodes, operative time (minutes), “upstaging” after surgery, blood loss during surgery (ml), and whether an anastomotic leak was present (yes/no). We defined “upstaging” as stage migration from clinical to pathologic stage, including only the nodal (N) stage, without a change in the tumor (T) stage. We defined our index date as the date of cancer diagnosis to encompass the entirety of oncologic care, including neoadjuvant therapy, at a patient’s initial clinical stage rather than the date of surgery and started with a patient’s pathologic stage.

### Statistical Analysis

Categorical patient demographics, clinical characteristics, and outcomes were compared using chi-square tests or Fisher’s exact tests for smaller populations. Non-normally distributed continuous variables, including age at diagnosis, operative time, and blood loss, were compared using nonparametric Wilcoxon rank-sum tests. A logistic regression analysis modeled the odds of obtaining at least one positive lymph node for D2 versus D1 LAD, with adjustment for age, clinical cancer stage, neoadjuvant treatment status, and CCI score.

The survival time for D1 versus D2 LAD during 5 years was assessed with Kaplan-Meier methods. Patients were followed from the date of cancer diagnosis until either the event of interest (death) or the end of the 5-year period. Patients lost to follow-up evaluation before the end of the 5-year period were censored at their last confirmed membership date. Additionally, patients without 5-years of follow-up evaluation by 31 March 2023, the last date of available mortality data, also were censored. Higher censorship was observed in the D2 group due to performance of this surgery with greater frequency in the later study years.

A Cox proportional hazards regression was used to explore the association between D1 versus D2 LAD and overall survival, with adjustment for relevant patient and clinical characteristics, including age, clinical cancer stage, treatment history, CCI score, and anastomotic leak status. The proportional hazards assumption was assessed with log-log graphs and Schoenfeld residuals.

All analyses were conducted with SAS version 9.4 (SAS Institute, New York, NY, USA). Statistical significance was set at a *p* value lower than 0.05. All statistical tests were two-tailed.

## Results

The study identified 155 adult patients with a Siewert II diagnosis during the study period from 1 January 2012 through 30 June 2022. Of the 155 patients, 129 (83%) were male, and 26 (17%) were female. Ages at the date of diagnosis ranged from 28 to 84 years (mean, 64.2 ± 11.0 years). Of the 155 patients, 115 (74%) underwent a D1 LAD, and 40 (26%) underwent a D2 LAD. The baseline patient characteristics (age, race/ethnicity, sex, BMI at diagnosis, CCI score, smoking history, and history of alcohol abuse) did not differ significantly between the Siewert II patients who underwent a D1 LAD and those who underwent a D2 LAD (Table 1).

**Table 1:** Baseline demographic and clinical characteristics of Siewert II patients stratified by D1 and D2 LAD

Characteristics	Total (*n* = 155) *n* (%)	D1 (*n* = 115) *n* (%)	D2 (*n* = 40) *n* (%)	*p* Value
*Age (years)*				0.125^a^
Median (IQR)	66.0 (58.0–72.0)	66.0 (59.0–73.0)	63.5 (55.0–70.5)	
Mean ± SD	64.2 ± 11.0	65.2 ± 10.3	61.4 ± 12.8	
Min–max	28.0–84.0	30.0–83.0	28.0–84.0	
*Race/ethnicity*				0.241^b^
White	106 (68.4)	81 (70.4)	25 (62.5)	
African-American	6 (3.9)	6 (5.2)	0 (0.0)	
Hispanic	15 (9.7)	10 (8.7)	5 (12.5)	
Asian/Pacific Islander	22 (14.2)	13 (11.3)	9 (22.5)	
Other	6 (3.9)	5 (4.3)	1 (2.5)	
*Sex*				0.727
Female	26 (16.8)	20 (17.4)	6 (15.0)	
Male	129 (83.2)	95 (82.6)	34 (85.0)	
*BMI at diagnosis (kg/m*^*2*^*)*				0.391
<25	48 (31.0)	36 (31.3)	12 (30.0)	
25.0–29.9	65 (41.9)	51 (44.3)	14 (35.0)	
≥30	42 (27.1)	28 (24.3)	14 (35.0)	
*CCI score*				0.527
0–3	105 (67.7)	80 (69.6)	25 (62.5)	
4–5	24 (15.5)	18 (15.7)	6 (15.0)	
6+	26 (16.8)	17 (14.8)	9 (22.5)	
*History of smoking*				0.938
Never	69 (44.5)	50 (43.5)	19 (47.5)	
Yes	24 (27.9)	18 (27.7)	6 (28.6)	
Former smoker	62 (72.1)	47 (72.3)	15 (71.4)	
*History of alcohol abuse*				1.000^b^
No	152 (98.1)	113 (98.3)	39 (97.5)	
Yes	3 (1.9)	2 (1.7)	1 (2.5)	
*Prior diagnostic laparoscopy*				**< 0.001**
No	68 (43.9)	64 (55.7)	4 (10.0)	
Yes	87 (56.1)	51 (44.3)	36 (90.0)	
*Neoadjuvant radiation*				**< 0.001**
No	56 (36.1)	29 (25.2)	27 (67.5)	
Yes	99 (63.9)	86 (74.8)	13 (32.5)	
*Neoadjuvant chemo*				0.186^b^
No	13 (8.4)	12 (10.4)	1 (2.5)	
Yes	142 (91.6)	103 (89.6)	39 (97.5)	
*Adjuvant radiation*				0.283^b^
No	145 (93.5)	109 (94.8)	36 (90.0)	
Yes	10 (6.5)	6 (5.2)	4 (10.0)	
*Adjuvant chemotherapy*				**0.002**
No	97 (62.6)	80 (69.6)	17 (42.5)	
Yes	58 (37.4)	35 (30.4)	23 (57.5)	
*Histology*				0.323^b^
Adenocarcinoma	145 (93.5)	106 (92.2)	39 (97.5)	
Squamous cell	7 (4.5)	7 (6.1)		
Other	3 (1.9)	2 (1.7)	1 (2.5)	
*Clinical stage*				0.076
I	8 (5.2)	8 (7.0)		
II	27 (17.4)	23 (20.0)	4 (10.0)	
III	88 (56.8)	64 (55.7)	24 (60.0)	
IV	32 (20.6)	20 (17.4)	12 (30.0)	

LAD, lymphadenectomy; IQR, interquartile range; SD, standard deviation; BMI, body mass index; CCI, Charlson Comorbidity Index

^a^Wilcoxon two-sample test was used for continuous variables

^b^Fisher’s exact test was used due to small cell sizes. Otherwise, the chi-square test was used

**Fig. 1:**
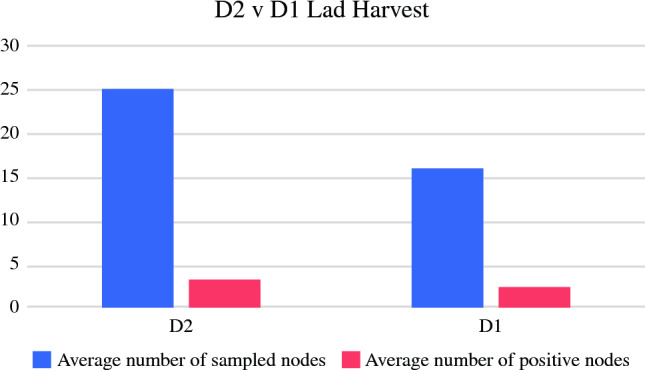
D2 versus D1 lymphadenectomy (LAD) harvest. The patients with a D2 LAD had more lymph nodes harvested than the patients with a D1 LAD (average 25.1 vs 15.7), but a similar average number of positive nodes (average, 2.8 vs 2.1)

Both the patients undergoing a D1 LAD and those undergoing a D2 LAD most often had clinical stage III disease at the time of their diagnosis (Table 1) and pathologic stage III disease (Table 2) after surgical resection. The patients with a D2 LAD had more than 15 lymph nodes harvested more frequently than the patients with D1 LAD (83% vs 48%; *p* < 0.001), but the mean number of positive nodes did not differ significantly (2.8 ± 5.2 D2 vs 2.1 ± 4.2 D1; *p* = 0.355 (Table 2; Fig. 1).

**Table 2:** Outcomes for Siewert II patients who underwent D1 versus D2 LAD

Outcomes	Total (*n* = 155) *n* (%)	D1 (*n* = 115) *n* (%)	D2 (*n* = 40) *n* (%)	*p* value
*Upstaged*				0.267^a^
No	136 (87.7)	103 (89.6)	33 (82.5)	
Yes	19 (12.3)	12 (10.4)	7 (17.5)	
*Operation time (min)*				< 0.001^b^
Median (IQR)	270.0 (211.0–378.0)	244.0 (201.0–335.0)	362.0 (290.0–407.0)	
Mean ± SD	297.8 ± 114.0	278.4 ± 114.2	353.7 ± 93.9	
Min–max	119.0–629.0	119.0–629.0	146.0–543.0	
*Blood loss (ml)*				0.087^b^
Median (IQR)	100.0 (50.0–150.0)	100.0 (50.0–150.0)	75.0 (25.0–150.0)	
Mean ± SD	148.0 ± 287.9	168.1 ± 329.6	90.4 ± 71.2	
Min–max	5.0–3000.0	10.0–3000.0	5.0–250.0	
*Anastomotic leak*				0.381^a^
No	138 (89.0)	104 (90.4)	34 (85.0)	
Yes	17 (11.0)	11 (9.6)	6 (15.0)	
*Pathologic stage*				0.119
0/Pathologic complete response	25 (16.1)	18 (15.7)	7 (17.5)	
I	28 (18.1)	24 (20.9)	4 (10.0)	
II	32 (20.6)	27 (23.5)	5 (12.5)	
III	54 (34.8)	37 (32.2)	17 (42.5)	
IV	16 (10.3)	9 (7.8)	7 (17.5)	
*No. of nodes sampled*				< 0.001
<15	67 (43.2)	60 (52.2)	7 (17.5)	
15+	88 (56.8)	55 (47.8)	33 (82.5)	
*No. of nodes positive*				0.355^b^
Median (IQR)	0.0 (0.0–3.0)	0.0 (0.0–3.0)	1.0 (0.0–3.0)	
Mean ± SD	2.3 ± 4.5	2.1 ± 4.2	2.8 ± 5.2	
Min–max	0.0–32.0	0.0–32.0	0.0–22.0	

LAD, lymphadenectomy; IQR, interquartile range; SD, standard deviation

^a^Fisher’s exact was test used due to small cell sizes. Otherwise, chi-square test was used

^b^Wilcoxon two-sample test was used for continuous variables

In a multivariable logistic regression model, including patients treated with the transhiatal approach, no significant difference was demonstrated between D2 and D1 for obtaining at least one positive lymph node (adjusted odds ratio [aOR], 1.58; 95 % confidence interval [CI], 0.73–3.44; *p* = 0.246; Table 3). When the study excluded the nine patients who underwent the transhiatal approach, similar results were demonstrated, with no significant difference between D2 and D1 for obtaining at least one positive lymph node (aOR, 1.26; 95% CI, 0.55–2.93; *p* = 0.586; Table S1), The patients with a D2 LAD had a median operative time approximately 2 h longer than those who had a D1 LAD (362 vs 244 min; *p* < 0.001). No significant difference was observed between the D2 and D1 LAD patients in the proportion of patients upstaged (18% vs 10%, respectively; *p* = 0.26; Table 2).

**Table 3:** Logistic regression results modeling that obtained one or more positive lymph nodes (*n* = 72) among 155 D1 and D2 patients^a^

Model variable	aOR (95 % CI)	*p* Value
D2 vs D1	1.58 (0.73–3.44)	0.246
Clinical stage	1.76 (1.06–2.93)	0.029
Age	0.97 (0.94–1.01)	0.094
Neoadjuvant treatment (Y vs N)	0.20 (0.05–0.84)	0.027
CCI score 4+ vs 0–3	0.80 (0.38–1.70)	0.560

aOR, adjusted odds ratio; CI, confidence interval; CCI, Charlson Comorbidity Index

^a^The 9 patients treated with a transhiatal surgical approach were included in the analysis

**Fig. 2:**
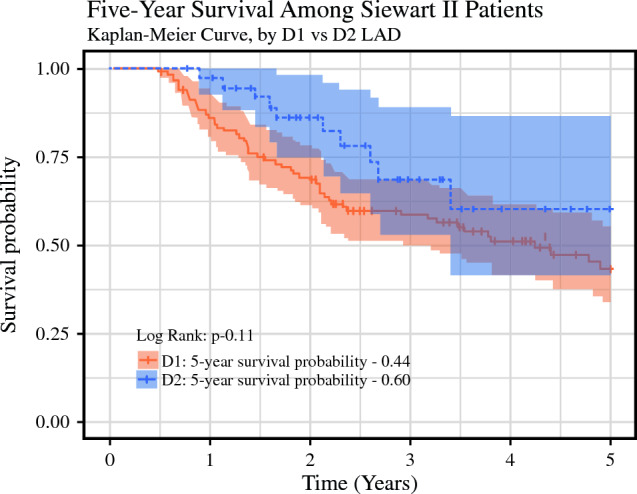
The 5-year Kaplan-Meier survival curve for Siewert II patients who underwent D1 versus D2 lymphadenectomy (LAD)

Kaplan-Meier analysis did not show a significant difference in 5-year overall survival probabilities between the D2 and D1 LAD patients (0.60 D2 vs 0.44 D1; *p* = 0.11; Fig. 2). In the Kaplan-Meier analysis, 38 D1 patients (33%) versus 29 D2 patients (73%) were censored without 5 full years of follow-up evaluation, and 55 D1 patients (48%) versus 10 D2 patients (25%) experienced the event of interest (death) during the 5 years.

In a multivariable Cox regression model, with adjustment for clinical stage, anastomotic leak (yes/no), neoadjuvant therapy (chemotherapy and/or radiation [yes/no]), age, and CCI score, no significant difference in overall survival was observed for the patients undergoing a D2 versus D1 LAD (adjusted hazard ratio [aHR], 0.52; 95% CI, 0.25–1.00; *p* = 0.067). A statistically significant overall survival difference in clinical staging was observed (*p* = 0.016; Table 4). Regarding the impact of extensive lymphadenectomy on locoregional, D1 and D2 LAD did not differ significantly in terms of 3-year recurrence-free survival (*p* = 0.11; Fig. S1).

**Table 4:** Multivariable Cox regression analysis of overall survival from the date of diagnosis among Siewert II patients who underwent D1 vs D2 LAD

Model variable	aHR (95 % CI)	*p* Value
*LAD surgery*		
D1	Reference	
D2	0.52 (0.25–1.00)	0.067
Clinical stage (continuous)	1.61 (1.10–2.38)	0.016
*Anastomotic leak*		
No	Reference	
Yes	1.53 (0.66–3.07)	0.273
Age (continuous)	1.01 (0.99–1.04)	0.291
*CCI*		
0–3	Reference	
4+	0.76 (0.42–1.33)	0.342
*Neoadjuvant treatment*^*a*^		
No	Reference	
Yes	0.62 (0.26–1.73)	0.312

LAD, lymphadenectomy; aHR, adjusted hazard ratio; CCI, Charlson Comorbidity Index

^a^Chemotherapy and/or radiology

**Fig. 3:**
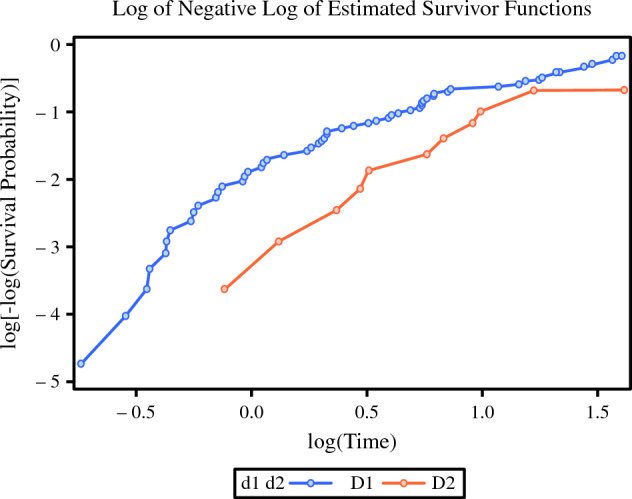
Log minus log function survival curve of D1 versus D2 lymphadenectomy (LAD)

The proportional hazards assumption was not violated in this multivariable Cox regression model. A log-log plot graphically demonstrated no significant departures from the proportional hazards assumption for surgery type (D1 vs D2 LAD; Fig. 3). Additionally, plots of scaled Schoenfeld residuals and a global test of the Schoenfeld residuals for a slope of 0 (*p* = 0.28; chi-square, 9.78; df, 8) supported the proportional hazards assumption (Fig. S2).

## Discussion

Current literature regarding the extent of LAD in GEJ cancer remains controversial, with the majority of current data specific to gastric cancer or GEJ cancer with curative-intent gastrectomy.^10^ This is the first study to detail the outcomes of Siewert II GEJ tumors treated with D1 versus D2 LAD via a minimally invasive trans-thoracic, Ivor-Lewis esophagectomy approach.

The recent AJCC definition of Siewert II tumors deemed as esophageal tumors has important surgical and therapy implications that distinguish them from Siewert III or gastric tumors.^7^ This is an important distinction to make because the benefits of D2 LAD leading to decreased locoregional recurrence and disease-specific survival as seen in the largest prospective, randomized trial were limited to patients with GEJ tumors who underwent gastrectomy and not an esophagectomy with curative intent.^9^

In terms of obtaining an adequate LAD, Ivor-Lewis esophagectomy has been shown to adequately obtain thoracic and abdominal LN with feasibility to perform a D2 dissection at the discretion of the surgeon.^12,13^ In addition to the differences in surgical management seen in our study compared with prior literature, major inconstancies exist in the optimal neoadjuvant and/or adjuvant therapies for Siewert II tumors. In our study, the Siewert II patients undergoing D1 LAD were more likely to have undergone neoadjuvant and concurrent chemoradiation (75 %) than those who underwent D2 LAD (33 %). Additionally, the patients undergoing D2 LAD were more likely to receive adjuvant chemotherapy (58 %) than those who underwent D1 LAD (30 %).

Further adding to the complexity of Siewert II tumor management, our patients were left to the discretion of the multidisciplinary tumor board review whether to use chemoradiotherapy for esophageal cancer as in the surgery study (CROSS) or to use perioperative chemotherapy with fluorouracil plus leucovorin, oxaliplatin, and docetaxel versus fluorouracil or capecitabine plus cisplatin and epirubicin for locally advanced, resectable gastric or gastro-oesophageal junction adenocarcinoma as in both the FLOT4 trial and the Medical Research Council Adjuvant Gastric Infusional Chemotherapy (MAGIC) trial.^14–16^ We found that the patients who underwent D1 dissection were more likely to receive neoadjuvant chemoradiation and thus follow the CROSS study regimen, whereas the patients who underwent D2 were more likely to receive treatment similar to gastric cancer management and to be treated with perioperative chemotherapy instead of chemoradiotherapy, in accordance with the FLOT4 trial, with the addition of staging diagnostic laparoscopy.^16^

Although it is difficult to say that the addition of neoadjuvant radiotherapy allows for less extensive LAD via D1 or that D2 may increase nodal yield to necessitate adjuvant chemotherapy, these differences in surgical management are important to note because current prospective randomized studies have not demonstrated a standardized approach, which may be ultimately influenced by differences in current oncologic therapy.

Although D2 LAD has the theoretical advantage of a more extensive LN harvest to influence pathologic staging, its clinical significance in terms of improving overall survival is controversial.^8,17^ As expected, the Siewert II patients who underwent D1 LAD had a smaller nodal harvest than the patients who underwent D2 LAD, with 48 % of the D1 LAD procedures yielding more than 15 LNs versus 83 % of the D2 LAD procedures (*p* < 0.001). However, according to the AJCC, accurate staging of GEJ cancer is defined by the number of positive LNs in addition to R0 resection.^1,7^ When LN positivity was evaluated between those receiving D1 LAD and those treated with D2 LAD, the two groups did not differ significantly (Table 2; Fig. 1). Additionally, D2 LAD had minimal to no impact on the Siewert II patients in terms of changing management because the patients who were clinically stage III or IVa remained in their respective stages. Stage migration occurred in 17.5% (*n* = 7) of the Siewert II patients undergoing D2, with six patients migrating from stage III to stage IV, and one patient migrating from stage II to stage IV.

The benefits seen with a larger nodal harvest for pathologic staging in prior studies has been largely limited to gastric cancer and has not been well-studied for GEJ cancer with an intent-to-cure esophagectomy. Although extended LAD with D2 may yield a larger LN harvest, its ability to influence stage migration and ultimately guide therapy is questionable because extended LAD did not lead to the capture of more malignant cells in Siewert II tumors.

Although multiple studies exist regarding the increasing Western popularity of D2 LAD in the management of GEJ cancer, the benefits have been limited to patients undergoing intent-to cure gastrectomies.^8,9,18^ Additionally, in multiple prospective, randomized trials, patients undergoing D2 LAD had greater perioperative morbidity and mortality with no change in overall survival for patients with GEJ cancer.^9,18^

Although D2 LAD has gained more interest recently, it remains an additional technically demanding step, ultimately increasing the patient’s time under anesthesia.^8^ Evaluation of operative time showed that Siewert patients with a D2 LAD had a median operative time approximately 2 h longer than patients with D1 LAD. Longer operative time and time under anesthesia for esophagectomies have been known to lead to postoperative complications such has increased incidences of pneumonia, length of stay, unplanned re-intubation, and mortality.^19^ These increased short-term complications with D2 LAD may be outweighed by the improved long-term benefits of decreased locoregional recurrence and gastric cancer-related deaths, although this remains controversial.^9^ Several studies have demonstrated long-term benefit from D2 LAD performed for gastric cancer, but no study has demonstrated improved overall survival.^8,9,18^

In our study, after adjustment for patient and clinical characteristics, overall survival did not differ significantly between the Siewert II patients undergoing D1 LAD and those treated with D2 LAD. Similar to other studies for gastric cancer, D2 LAD did not confer a significant difference in survival while adding additional 2 h of operating time when performed as an intent-to-cure esophagectomy. As expected, increasing clinical stage at diagnosis rather than performance of a D1 or D2 LAD demonstrated an overall survival difference that was statistically significant in Cox multivariable regression. Although improvements in surgical technique and medical therapy have made D2 LAD more feasible for the treatment of gastric cancer, its routine use in Siewert II esophagectomies remains controversial, particularly while similar concerns of lack of overall survival benefit still exist.

This study had several limitations. The study was retrospective in nature and limited to a single tertiary center. However, this is the largest of all the regionalized referral centers for esophagectomy in the entire region, routinely performing more than 40 minimally invasive esophagectomies (MIE) per year. Specific chemoradiation regimens were difficult to characterize accurately as radiotherapy, which is part of our center’s treatment algorithm. The difficulty was because outsourcing of radiation oncology referrals was dependent on the geographic region in which the patient resided.

Additionally, granular data regarding dosing, timing, and number of chemotherapy cycles were difficult to characterize, which may have led to inaccurate characterization of adjuvant therapy. In the modeling for overall survival, the diagnosis in the D2 LAD group occurred more often between 2018 and 2022 and therefore were more often censored in the model without a full 5 years of follow-up time available. Higher levels of censorship may have led to wider confidence intervals, limiting our findings.

The convergence of the log-log plot (Fig. 3) demonstrates that the relative hazards of the D1 and D2 LADs approached each other as time progressed. Similarly, individual scaled Schoenfeld residual tests (Fig. S2) indicate potential time-varying effects for D1 versus D2 (*p* = 0.07) and clinical stage (*p* = 0.06). Further exploration is suggested to examine whether this is due to a decreasing effect over time or to unbalanced censorship.

It also is important to note the operative time discrepancy between the D1 and D2 groups. TheD2 LAD requires more meticulous dissection, and our study demonstrated an operative time difference of approximately 2 h. This likely was due to the addition of a separate surgical team to perform the D2 dissection according to the protocol of our institution, in which different surgical trays and operative room turnover may account for this additional time difference, perhaps skewing the results regarding the operative disadvantage of performing a D2 versus D1 dissection. To further mitigate this discrepancy, we plan to convert all laparoscopic trays to one centralized universal tray set with the amount of the abdominal portion of the operation by different surgical teams consolidated from start to finish, with hopes of decreasing operative times and improving efficiency while maintaining similar outcomes.

In conclusion, despite the associated high mortality associated with GEJ cancer, few consensus guidelines exist regarding the optimal preoperative and operative management. Although D2 LAD may theoretically lead a larger lymph node harvest and may be warranted in select cases, its routine use for Siewert II tumors may not necessarily be mandatory because it did not increase the number of positive nodes and had no significant impact on survival.

## Supplementary Information

Below is the link to the electronic supplementary material.

Supplementary file1 (DOCX 13 kb)

Supplementary file2 (DOCX 32 kb)

Supplementary file3 (TIFF 1894 kb)

Supplementary file4 (TIFF 1894 kb)
