# Correction: The Impact of D2 Versus D1 Lymphadenectomy in Siewert II Gastroesophageal Junction (GEJ) Cancer

**DOI:** 10.1245/s10434-024-16179-8

**Published:** 2024-09-05

**Authors:** Nathan J. Alcasid, Deanna Fink, Kian C. Banks, Cynthia J. Susai, Katherine Barnes, Rachel Wile, Angela Sun, Ashish Patel, Simon Ashiku, Jeffrey B. Velotta

**Affiliations:** 1 Department of General Surgery, University of California, San Francisco-East Bay, Oakland, CA USA; 2 Division of Research, Kaiser Permanente Northern California, Oakland, CA USA; 3 Division of Thoracic Surgery, Department of Surgery, Kaiser Permanente Northern California, Oakland, CA USA

**Correction to: Ann Surg Oncol** 10.1245/s10434-024-15623-z

In the original online version of this article, there is an error in the last sentence of the results in the abstract. The correct sentence is as follows:

In Kaplan-Meier and multivariable Cox regression models, overall survival did not differ significantly between the patients undergoing D2 and those who had D1 (adjusted hazard ratio [aHR], 0.52; 95% confidence interval [CI], 0.25–1.00; p = 0.067).

The original article was corrected.

