# Supplementary material for: The Impact of D2 Versus D1 Lymphadenectomy in Siewert II Gastroesophageal Junction (GEJ) Cancer
Source: Ann Surg Oncol. 2024 Jul 30;31(12):8148–56. doi: 10.1245/s10434-024-15623-z (PMC11467080; doi:10.1245/s10434-024-15623-z)
Supplement: Supplementary file 1 — Supplementary file1 (DOCX 13 kb) [file 10434_2024_15623_MOESM1_ESM.docx]

**Supplemental Table 1:** Logistic regression results modeling obtaining 1+ positive lymph nodes (n=65) among 146 D1 and D2 patients

| **Model variable** | **Adjusted Odds Ratio (95% CI)** | **P value** |
| --- | --- | --- |
| D2 v D1 | 1.26 (0.55-2.93) | 0.586 |
| Clinical stage | 1.73 (1.02-2.94) | 0.043 |
| Age | 0.97 (0.93-1.00) | 0.052 |
| Neoadjuvant treatment Y v N | 0.23 (0.05-0.99) | 0.049 |
| CCI score 4+ v 0-3 | 0.65 (0.30-1.43) | 0.285 |

*Note: the 9 patients with transhiatal surgical approach were excluded from analysis
