# Supplementary figures and images for: The Impact of D2 Versus D1 Lymphadenectomy in Siewert II Gastroesophageal Junction (GEJ) Cancer
Source: Ann Surg Oncol. 2024 Jul 30;31(12):8148–56. doi: 10.1245/s10434-024-15623-z (PMC11467080; doi:10.1245/s10434-024-15623-z)

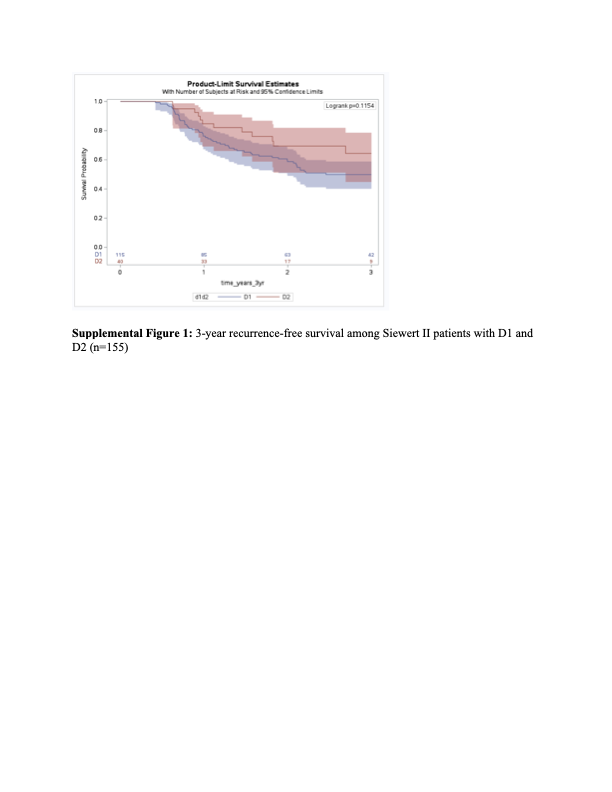

Supplement: Supplementary file 3 — Supplementary file3 (TIFF 1894 kb) [file 10434_2024_15623_MOESM3_ESM.tiff]

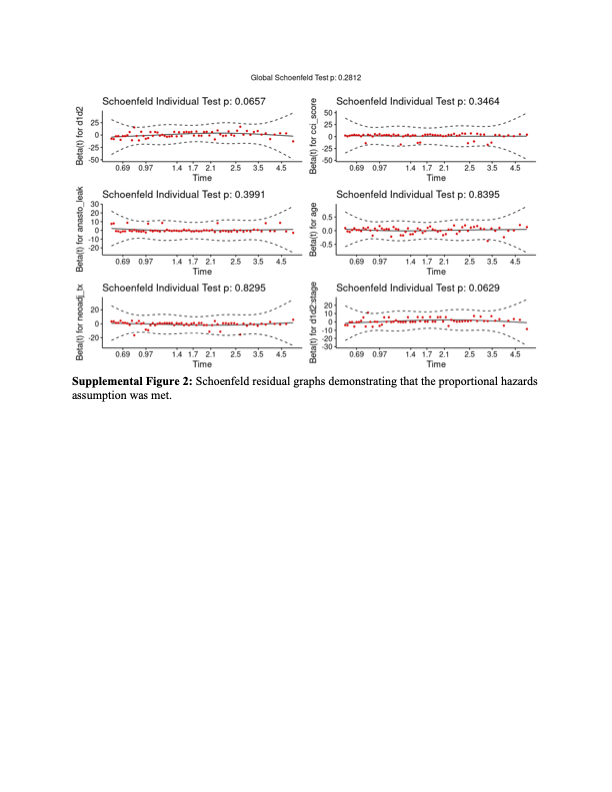

Supplement: Supplementary file 4 — Supplementary file4 (TIFF 1894 kb) [file 10434_2024_15623_MOESM4_ESM.tiff]
